# Phosphodiesterase 4 (PDE 4) Inhibition Reduces Ischemia–Reperfusion-Induced Leucocyte Infiltration, Apoptosis and Mitochondrial Fission Markers in Mice Skeletal Muscles Four Hours After Ischemia Onset

**DOI:** 10.3390/muscles5010019

**Published:** 2026-03-03

**Authors:** Anne-Laure Charles, Liliane Tetsi, Giulia Quiring, Cindy Barnig, Margherita Giannini, Alain Meyer, Anne Lejay, Claire Lugnier, Bernard Geny

**Affiliations:** 1 UR 3072, “Mitochondria, Oxidative Stress and Muscle Plasticity”, Biomedicine Research Center of Strasbourg (CRBS), Faculty of Medicine, University of Strasbourg, 67081 Strasbourg, France; anne.laure.charles@unistra.fr (A.-L.C.); gemmelere@yahoo.fr (L.T.); giulia.quiring@etu.unistra.fr (G.Q.); cbarnig@chu-besancon.fr (C.B.); margherita.giannini@chru-strasbourg.fr (M.G.); alain.meyer1@chru-strasbourg.fr (A.M.); anne.lejay@chru-strasbourg.fr (A.L.); claire.lugnier@unistra.fr (C.L.); 2 Department of Physiology and Functional Explorations, University Hospital of Strasbourg, 67000 Strasbourg, France; 3 Department of Vascular Surgery, University Hospital of Strasbourg, 67000 Strasbourg, France

**Keywords:** muscle, ischemia–reperfusion, cyclic nucleotide phosphodiesterase (PDE) inhibition, PDE 4, inflammation, leucocytes, apoptosis, mitochondria, fission, oxidative stress

## Abstract

Peripheral arterial disease is a leading cause of amputation and/or death worldwide. Phosphodiesterase 4 (PDE 4) inhibitors demonstrated beneficial effects in ischemia–reperfusion (IR) settings, but whether PDE 4 inhibition protects skeletal muscle against IR deleterious effects is unknown. We therefore performed limb IR (two hours each) in twenty-one male Swiss mice (12–16-week-old) treated or not with Rolipram (1 mg/kg i.p. 30 min before ischemia and 5 min before reperfusion). The muscles were analyzed 4 h after the onset of ischemia. IR significantly increased leucocyte infiltration (93.13 ± 6.886 vs. 150.1 ± 18.38 cells/mg of muscle, *p* < 0.05) and apoptosis (Bax/Bcl2 ratio, +239%, *p* < 0.05), together with enhanced mitochondrial fission transcripts (+224% for Drp1, *p* < 0.01 and +368%, *p* < 0.0001 for Fis1), and decreased mitochondrial respiration and antioxidant defense. PDE 4 inhibition reduced leucocyte infiltration (150.1 ± 18.38 vs. 55.58 ± 13.83; *p* < 0.01) and apoptosis (+67%, NS) in association with reduced fission markers (+91% for Drp 1 and +111%, *p* < 0.05, for Fis 1). Muscle mitochondrial respiration did not improve. In conclusion, PDE 4 inhibition using Rolipram partly protected skeletal muscles against IR-induced deleterious effects. These data support further studies investigating the usefulness of leucocytes modulation in lower-limb IR and a potential beneficial effect of PDE 4 inhibition in peripheral arterial disease.

## 1. Introduction

Cyclic nucleotide phosphodiesterase families (PDEs) participate in many cell functions through their regulation of cAMP and/or cGMP intracellular signaling pathways. The PDE4 family specifically hydrolyses cAMP and modulates fundamental functions allowing therapeutic applications [1]. Particularly, Rolipram is a PDE4 inhibitor known for its anti-inflammatory properties, mainly in diseases such as asthma and chronic obstructive pulmonary disease (COPD), where it decreases the production of TNF-alpha and other proinflammatory cytokines [2,3,4]. Rolipram also reduced polynuclear leukocytes (PMNs) recruitment and activation in experimental models of inflammation [5]. Further, Rolipram protects several organs from severe injuries; thus, it reduced oxidative renal injury in acute pyelonephritis in rats [6]. In the setting of ischemia–reperfusion, Rolipram had protective effects on the intestine [7], the stomach [8], the kidney [9] the brain [10,11], the testis [12] and the ovaries [13]. Considering skeletal muscle, combined PDE 4 and 5 inhibition had anti-fibrotic effects on the gastrocnemius in mdx mice [14]. Accordingly, PDE 4 is expressed in skeletal muscle [14,15,16]. In view of these data, whether PDE 4 inhibition might also present beneficial effects on skeletal muscle submitted to ischemia–reperfusion deserves to be investigated.

Indeed, peripheral arterial disease characterized by lower-limb ischemia–reperfusion in humans is a public health issue, characterized by bad prognosis both locally (amputation) and on the systemic level (ranging from decreased quality of life to death). The pathophysiology of lower-limb ischemia–reperfusion includes muscle mitochondrial dysfunction, increased oxidative stress, and inflammation. During ischemia, less ATP is generated, leading to cellular edema. Reperfusion can prevent irreversible damage, but it might also be deleterious through the generation of oxidative stress. Indeed, the reactive oxygen species (ROS) produced can exceed the cellular antioxidant defenses. Production of free radicals will impair the mitochondrial respiratory chain, generating more ROS and potentially inducing lipid peroxidation, protein oxidation, DNA mutations and opening of the mitochondrial permeability transition pore, leading to apoptosis [17,18,19,20]. Accordingly, mitochondria are the main powerhouse of the cells and modulate cell death and oxidative stress resulting from increased reactive oxygen species production and/or decreased antioxidant defense. Thus, besides revascularization and exercise, depending on the severity of peripheral arterial disease, therapeutic approaches aiming to protect and even to replace impaired muscles mitochondria are promising [21,22,23,24,25].

Moreover, similarly to observations during chronic disease or aging [26], ischemia and reperfusion trigger a sterile immune response involving signaling events through pattern-recognition molecules such as Toll-like receptors. This allows recruitment and activation of immune cells of the innate and adaptive immune system and activation of the complement system.

During the early phase of reperfusion, innate immune cells dominate the cellular composition of the infiltrates. The functional contributions of these innate immune cells, mainly neutrophils, remain to be determined, but leucocytes may contribute to a pathological activation of inflammation and promote uncontrolled inflammation and collateral tissue injury; interestingly, previous data demonstrated that a reduction in leucocytes decreased muscle injury in several experimental settings including IR [27,28,29,30,31,32,33,34].

The aim of this study was to investigate whether PDE4 inhibition might protect skeletal muscles against ischemia–reperfusion-induced deleterious effects by reducing leucocytes infiltration, apoptosis and mitochondrial dysfunctions. We therefore determined leucocyte recruitment and apoptosis, together with oxidative stress and mitochondrial functions at the cellular, mitochondrial and transcripts levels in ischemic and non-ischemic skeletal muscles using a well-characterized experimental model of lower-limb ischemia–reperfusion.

## 2. Results

### 2.1. PDE 4 Inhibition Reduced Ischemia–Reperfusion-Induced Leucocyte Infiltration

Since PDE 4 inhibition presents with anti-inflammatory properties, we assessed leucocyte infiltration. Indeed, on one hand, immune cells are required to clear cellular debris and aid in the regenerative response. On the other hand, excessive inflammation delays cells and tissue reparations.

**Figure 1:**
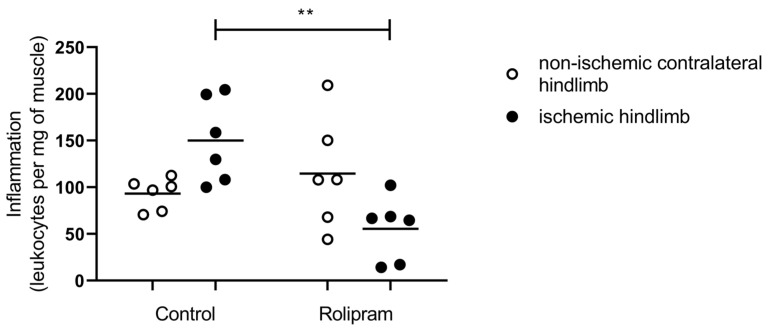
Rolipram reduced IR-related leucocytes infiltration. For both control and Rolipram groups, left white bar, non-ischemic contralateral hindlimb; right black bar, ischemic hindlimb. Results areexpressed as means ± SEM. **: *p* < 0.01. n = 6 per group.

In the control group, total leukocytes tended to be increased in the ischemic quadriceps muscle as compared to the non-ischemic muscle (+61%, 93.13 ± 6.89 vs. 150.1 ± 18.38 cells/mg of muscle; Figure 1).

Rolipram treatment reduced leucocyte infiltration. Thus, the number of leucocytes was significantly lower after IR in the ischemic leg with Rolipram as compared to the ischemic leg without Rolipram (150.1 ± 18.38 vs. 55.58 ± 13.83; *p* < 0.01). As compared to the non-ischemic contralateral quadriceps muscle, such a decrease tended to reach statistical significance (114 ± 24.09 vs. 55.58 ± 13.83 cells/mg of muscle, *p* = 0.09).

### 2.2. PDE 4 Inhibition Reduced Ischemia–Reperfusion-Induced Apoptosis

**Figure 2:**
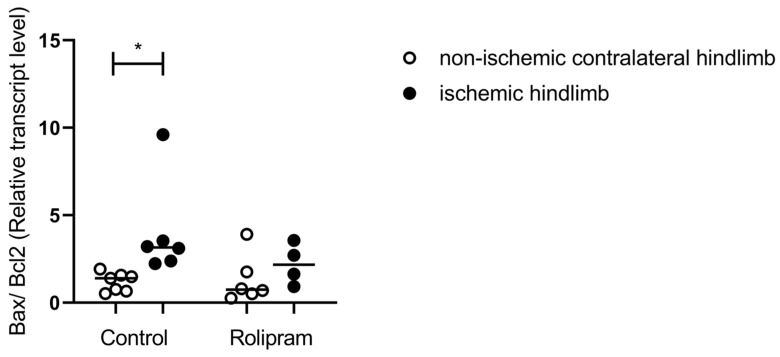
Rolipram blunted the IR-related Bax/Bcl2 ratio increase in gastrocnemius. Transcripts of Bax/Bcl2. n = 4 for ischemic Rolipram group and n = 6 for the other groups. For both control and Rolipram groups, left white circles correspond to the non-ischemic hindlimb; right black circles correspond to the ischemic hindlimb. Results are expressed as means ± SEM. *: *p* < 0.05.

Apoptosis is a well-known pathway leading to cell death and it is involved in ischemia–reperfusion-related deleterious effects in muscles. We therefore investigated whether PDE 4 inhibition might modulate apoptosis in skeletal muscles. The RNA transcripts encoding for apoptosis (Bax/Bcl2 ratio) showed a significant increase after ischemia–reperfusion in the control group +239% (1.18 ± 0.20 vs. 4.00 ± 1.14, *p* < 0.05). In the Rolipram group, the small increase observed was not significant +67% (1.32 ± 0.56 vs. 2.20 ± 0.58) (Figure 2).

### 2.3. PDE 4 Reduced Mitochondrial Fission Transcripts Without Changes in Mitochondrial Respiration and Calcium Retention Capacity

Mitochondrial dynamics entails fission and fusion and appears interesting to study knowing its role in modulating mitochondrial morphology and function, including energetic metabolism.

**Figure 3:**
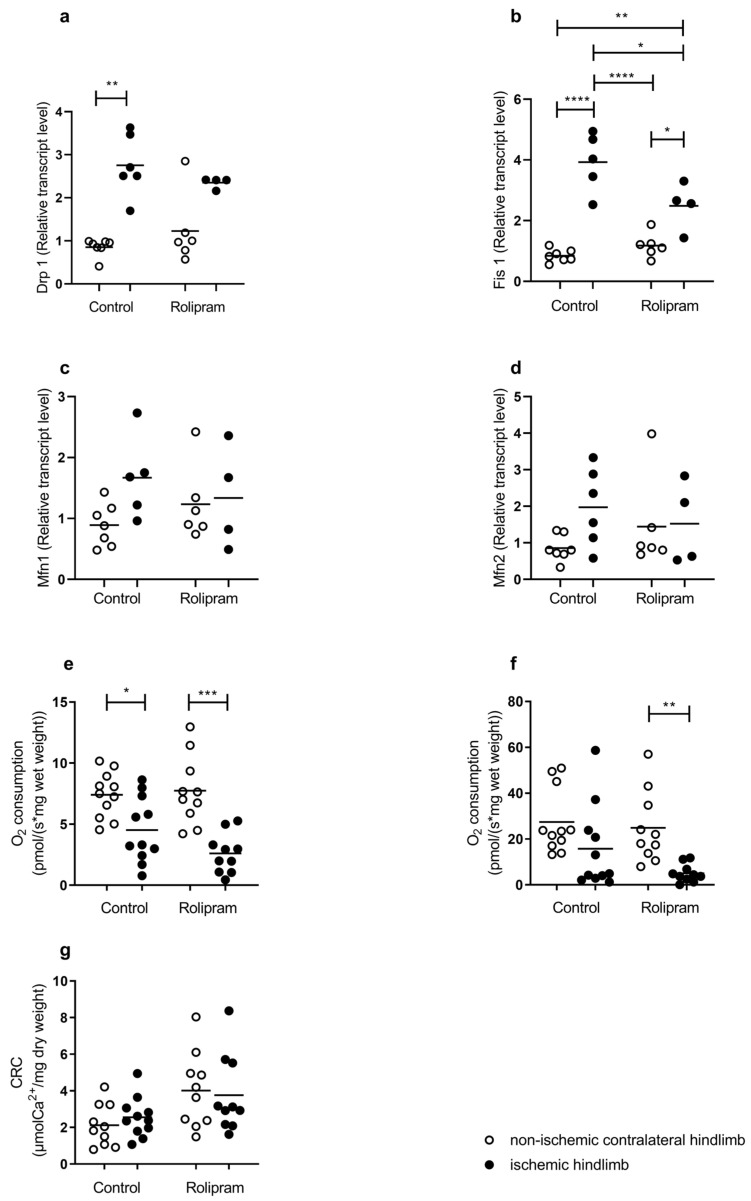
Mitochondrial fission transcripts, respiration and calcium retention capacity. (**a**,**b**) Transcripts of mitochondrial fission in ischemic and non-ischemic gastrocnemius muscles (**a**) Drp 1, (**b**) Fis 1 treated or not with Rolipram. Fis: mitochondrial fission protein, Drp1: dynamin-related protein. n = 4 for ischemic Rolipram group and n = 6 for the other groups. (**c**,**d**) Transcripts of mitochondrial fusion in ischemic and non-ischemic gastrocnemius muscles (**c**) Mfn1, (**d**) Mfn2 treated or not with Rolipram. Mfn: mitofusin. (**e**,**f**) Mitochondrial respiration (n = 11 for control group, and n = 10 for Rolipram group); (**e**) CI leak. (**f**) OXHOS CI. CI leak characterized the non-phosphorylant respiration by complex I, OXPHOS. CI: oxidative phosphorylation by complex I. (**g**) Calcium retention capacity (CRC) (n = 10 per group). For both control and Rolipram groups, left white bar is non-ischemic contralateral hindlimb; right black bar is ischemic hindlimb. Results are expressed as means ± SEM. *: *p* < 0.05, **: *p* < 0.01, ***: *p* < 0.001, ****: *p* < 0.0001.

Mitochondrial fission is generally considered as a deleterious mechanism reducing mitochondrial ability to generate ATP. Mitochondrial fission transcripts Drp1 and Fis1 increased after IR. In the control group, Drp1 increased from 224% (0.85 ± 0.08 vs. 2.76 ± 0.29, *p* < 0.01), as did Fis1 (368%, 0.84 ± 0.08 vs. 3.93 ± 0.43, *p* < 0.0001). In the Rolipram group, the increase was more moderate (+91% for Drp 1, NS, +111%, *p* < 0.05, for Fis 1) (Figure 3a,b). Taken together, these results suggest that an IR-induced increase in mitochondrial fission might be blunted by Rolipram. Mitochondrial fusion is a mechanism potentially preventing IR-related injury on skeletal muscle. In this study, the transcripts of Mfn1 and Mfn2 were not modified significantly after IR. In the control group, Mfn1 tended to increase from 0.89 ± 0.35 to 1.67 ± 0.68 (Figure 3c) and Mfn2 from 0.85 ± 0.36 to 1.97 ± 1.06 (Figure 3c,d). In the Rolipram group, the changes were similar for both transcripts.

Mitochondrial respiration analysis allows the investigation of the functional role of mitochondria which is the main energy source of the cells. Depending on substrates use, we can determine mitochondrial respiration associated (with ADP) or not (without ADP) with oxidative phosphorylation leading to ATP production. CI leak is non-phosphorylant respiration by the complex I, obtained after glutamate and malate injection. OXPHOS CI corresponds to the oxidative phosphorylation obtained after the addition of ADP. In the control group, the CI leak was significantly reduced in ischemic hindlimbs (7.40 ± 0.56 vs. 4.52 ± 0.80 pmolO_2_/s/mg ww; *p* < 0.05) (Figure 3e). OXPHOS CI tended to decrease in the ischemic hindlimbs of the control group from 27.45 ± 4.25 to 15.74 ± 5.52 pmolO_2_/s/mg ww (Figure 3f). In the Rolipram-treated mice, both CI leak and OXPHOS CI decreased in the ischemic hindlimbs as compared to the contralateral limb (from 7.75 ± 0.89 vs. 2.60 ± 0.51 pmolO_2_/s/mg ww, *p* < 0.001 and from 24.88 ± 4.93 vs. 5.03 ± 1.23 pmolO_2_/s/mg ww, *p* < 0.01); Figure 3c,d).

Finally, in the control and Rolipram groups, ischemia–reperfusion did not significantly modify the calcium retention capacity of the muscle (Figure 3g).

### 2.4. Effects of Ischemia–Reperfusion and PDE 4 Inhibition on Oxidative Stress

#### 2.4.1. Reactive Oxygen Species (ROS) Production

**Figure 4:**
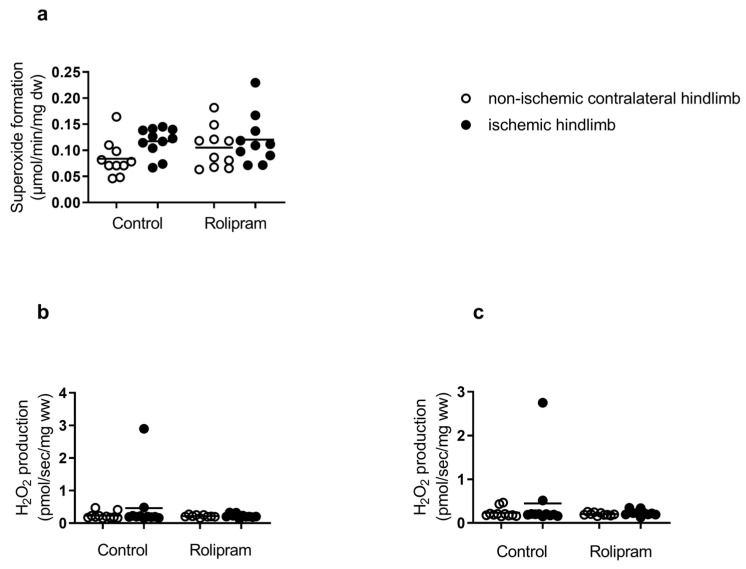
Reactive oxygen species (ROS) production in ischemic and non-ischemic gastrocnemius muscles in mice treated or not with Rolipram. (**a**) Superoxide level using electron paramagnetic resonance. (**b**,**c**) H_2_O_2_ production was measured simultaneously with mitochondrial respiration on oroboros with amplex red and HRP, with glutamate and malate addition (**b**) and ADP (**c**). For both control and Rolipram groups, left white bar is non-ischemic contralateral hindlimb; right black bar is ischemic hindlimb. Results are expressed as means ± SEM. n = 10 for control group and n = 10 for Rolipram group.

The production of superoxide anion tended to increase in the control ischemic limb (0.08 ± 0.01 vs. 0.12 ± 0.01 µmol/min/mg dw, +50%), but this increase did not reach statistical significance (Figure 4a). Similarly, hydrogen peroxide production tended to increase in the ischemic hindlimb compared to the contralateral hindlimb (in CI leak: 0.23 ± 0.03 vs. 0.46 ± 0.24 pmol/s/mg ww) and in OXPHOS CI: 0.23 ± 0.03 vs. 0.45 ± 0.23 pmol/s/mg ww, respectively) (Figure 4b,c).

In Rolipram-treated mice, we observed globally no significant change. Superoxide production was 0.10 ± 0.01 in the non-ischemic hindlimbs and 0.12 ± 0.01 µmol/min/mg dw in the ischemic hindlimbs, +20% (Figure 4a). There was also no change in hydrogen peroxide production in the ischemic hindlimb compared to the contralateral hindlimb (in CI leak: 0.22 ± 0.01 vs. 0.22 ± 0.02 pmol/s/mg ww) and in OXPHOS CI: 0.20 ± 0.01 vs. 0.23 ± 0.02 pmol/s/mg ww) (Figure 4b,c).

Thus, taken together, no significant change in reactive oxygen species was observed after IR, and Rolipram use did not modify such a result.

#### 2.4.2. Antioxidant Defense

**Figure 5:**
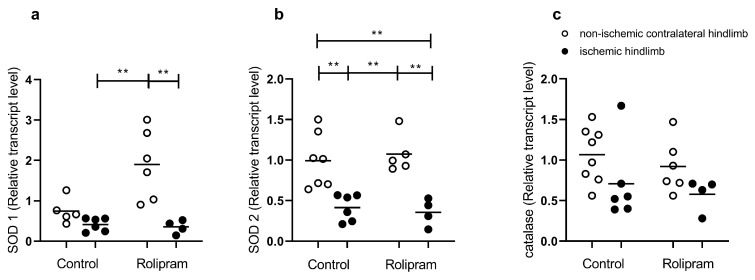
Transcripts of antioxidant pathways in ischemic and non-ischemic gastrocnemius muscles treated or not with Rolipram. (**a**) SOD1 (**b**) SOD2 (**c**) catalase treated or not with Rolipram. For both control and Rolipram groups, left white bar is non-ischemic contralateral hindlimb; right black bar is ischemic hindlimb. Results are expressed as means ± SEM. **: *p* < 0.01. SOD: superoxide dismutase. n = 4 for ischemic Rolipram group and n = 6 for the other groups.

Rolipram tended to increase SOD 1 transcript in the non-ischemic limb, but this failed to reach statistical significance. There was no difference in SOD 1 expression in the control group between uninjured and injured legs. The IR-induced SOD 1 decrease was significant when using Rolipram (1.90 ± 0.35 vs. 0.35 ± 0.08, *p* < 0.01, Figure 5a). IR-induced SOD 2 decreases were significant in the control group (0.99 ± 0.13 vs. 0.41 ± 0.07, *p* < 0.01, Figure 5b) and in the Rolipram group (1.07 ± 0.11 vs. 0.36 ± 0.08, *p* < 0.01, Figure 5b). RNA transcripts for catalase show no statistical difference in both control and Rolipram groups (Figure 5c).

## 3. Discussion

The main results of this study are that ischemia–reperfusion increased leucocyte recruitment and skeletal muscle apoptosis, in association with decreased antioxidant defense, enhanced mitochondrial fission and impaired mitochondrial respiration. PDE 4 inhibition with Rolipram reduced leucocyte infiltration, apoptosis and the mitochondrial fission markers (Drp1 and Fis1). Muscle mitochondrial respiration remained impaired after IR.

### 3.1. PDE 4 Inhibition Reduced Ischemia–Reperfusion-Induced Leucocyte Infiltration

Hindlimb IR is known to enhance leucocyte recruitment; accordingly, we previously observed significant leukocyte accumulation in the ischemic skeletal muscle [35]. Inflammation plays a critical role in the pathophysiology of IR injury, and during the early phase of reperfusion, neutrophils participate in acute IR injury by releasing inflammatory cytokines and by generating toxic oxygen metabolites in injured skeletal muscles [35,36,37,38,39,40].

Reducing such mechanisms can therefore be an interesting therapeutic approach. Indeed, a murine model of lipopolysaccharide (LPS)-induced lung injury showed that Rolipram inhibited neutrophil recruitment [41]. Further, PDE 4 inhibitors previously demonstrated anti-inflammatory effects, including in different settings of IR [5,7]. Thus, Rolipram prevented the development of gastric injury and inhibited an IR-induced increase in mucosal TNF-alpha content [8]. It also reduced brain edema together with decreased neutrophils recruitment and proinflammatory cytokine expression [10]. During ovarian IR, Rolipram also reduced inflammatory markers [13].

Accordingly, in our study, Rolipram significantly inhibited the leukocyte influx after reperfusion in ischemic skeletal muscle, supporting its efficacy as an anti-inflammatory agent and potential beneficial effects in the setting of skeletal muscle IR.

### 3.2. PDE 4 Inhibition Reduced Ischemia–Reperfusion-Induced Muscle Apoptosis

Apoptosis is observed in skeletal muscles subjected to IR, and Bax and Bcl-2 are two main cytoplasmic proteins that regulate apoptosis and are functionally opposed to each other. Precisely, the antiapoptotic Bcl-2 blocks death signal transmission by interfering with caspase activation. The Bax/Bcl-2 ratio determines the cell’s response to an apoptotic stimulus. This ratio, a good marker of apoptosis, increases during lower-limb ischemia–reperfusion. Mitochondrial calcium retention capacity is also considered as a surrogate of apoptosis and has been shown to be decreased in skeletal muscles subjected to IR [18,20].

In this study, the mitochondrial CRC was not impaired, limiting its ability to evaluate whether PDE 4 inhibition might reduce its implication in muscle apoptosis. Nevertheless, since Rolipram prevented mitochondrial apoptosis and neuron degeneration through regulation of the expression of Bax and Bcl-2 [42], we hypothesized that similar results might be obtained during muscle IR. Interestingly, PDE 4 inhibition using Rolipram also reduced the Bax/Bcl2 ratio and therefore apoptosis in the ischemic muscle. This is in accordance with previous results, including in diabetic rats where Rolipram decreased the activities of caspase-3 [33,34], and further supports the beneficial effect of PDE 4 inhibition in lower-limb ischemia–reperfusion.

### 3.3. Differential Effects of PDE 4 on Muscle Mitochondria

As expected, IR impaired mitochondrial dynamics, increasing significantly fission markers such as DRP1 and Fis1 transcripts. Mitochondrial dynamics plays a critical role in controlling mitochondrial morphology and energetic metabolism and has been shown to be modified in lower-limb IR [43,44,45,46,47]. Interestingly, DRP-1-mediated mitochondrial fission and apoptosis can be reduced by baicalin, suggesting a potential beneficial modulation of mitochondrial fission in skeletal muscle [43,44,45,46,47,48]. Rolipram reduced the IR-related enhancement of mitochondrial fission transcripts. Such an effect might participate in reducing apoptosis, observed after the PDE 4 inhibition tested in our experiment. Indeed, elevated mitochondrial fission is associated with increased cellular apoptosis and muscle fiber atrophy in disused muscle and sarcopenia. Nevertheless, like during aging, conflicting results are reported that challenge the proposed beneficial effects of decreased fission and of enhanced fusion in skeletal muscles, supporting further studies to investigate such issues [49].

IR decreased muscle mitochondrial respirations. This is consistent with previous reports using hindlimb IR models, secondary to aortic ligation or unilateral leg IR [18,19,20]. In this study, PDE 4 inhibition failed to protect mitochondria respiration after IR, the decrease being significant when considering OXPHOS respiration. A similar result was observed by Lange-Sperandio et al., demonstrating that PDE 4 delayed inflammation but failed to reduce injury in a model of obstructive nephropathy [50].

### 3.4. Effects of Rolipram on Reactive Oxygen Production and Antioxidant Defense

Oxidative stress participates in IR-related muscular damage, and we determined both ROS production and antioxidant defenses. In this study, ROS production was moderate, with superoxide anions and hydrogen peroxide tending to increase after IR. Nevertheless, IR globally reduced antioxidant enzymes transcripts. These data are in accordance with the literature, which usually shows increased ROS production together with impaired antioxidant defense. Accordingly, humans with peripheral arterial disease can present with increased skeletal muscle mitochondrial free radical production [51].

After Rolipram use, hydrogen peroxide increase tended to be lower in the ischemic limb while SOD 1 tended to increase in the non-ischemic limb. Although needing confirmation, these results might suggest a potential beneficial effect of PDE 4 inhibition as observed in other IR settings. However, in our study, Rolipram did not reduce oxidative stress.

## 4. Material and Methods

### 4.1. Animals

Male Swiss mice (12–16-week-old, Janvier, Le Genest-St-Isle, France) were housed with food and water *ad libitum* in a thermo-neutral environment at 22 ± 2 °C on a 12 h day/night cycle. This study obtained approval from the Regional Committee of Ethics in Animal Experimentation of Strasbourg (C.R.E.M.E.A.S) and the Ministry of Higher Education and Research (CREMEAS no. 2018022716192465v3, 25th October 2018).

### 4.2. Study Design

**Figure 6:**
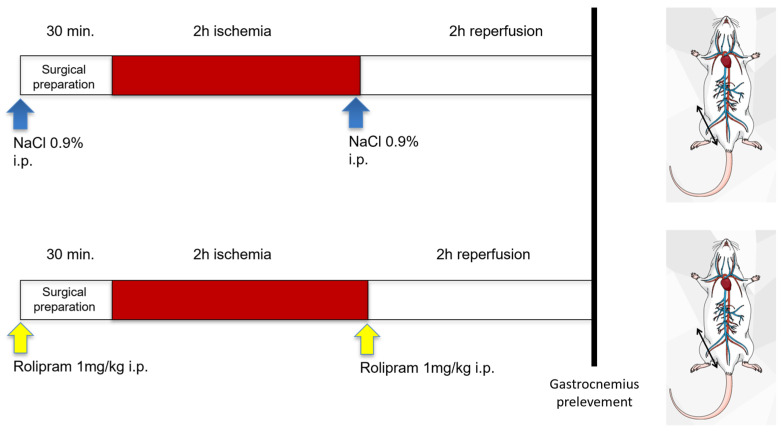
Experimental design. Animals underwent 2 h of unilateral hindlimb tourniquet ischemia (red bar), followed by 2 h of reperfusion. The muscles were therefore analyzed 4 h after the onset of ischemia. They received two intraperitoneal injections of either NaCl 0.9% (5 µL/g), or Rolipram (1 mg/kg), 30 min before surgery and 5 min before starting reperfusion. The non-ischemic hindlimb served as control.

All mice were subjected to 2 h ischemia through a tourniquet placed on the right hindlimb, followed by 2 h reperfusion (Figure 6). The left non-ischemic hindlimb served as a control, since previous data demonstrated that unilateral hindlimb ischemia did not significantly affect the contralateral hindlimb. Thirty minutes before ischemia, and 5 min before reperfusion, sham mice (n = 11) received intraperitoneal NaCl 0.9% (5 µL/g), and Rolipram mice (n = 10) received intraperitoneal NaCl 0.9% (1 mg/kg).

Placed in a hermetic anesthetic induction cage, mice were ventilated with a gas mixture of 4% isoflurane (AERRANE^®^, BAXTER S.A.S., Lessines, Belgium) and oxygen. Then, they were placed on heating blankets (MINERVE^®^, Esternay, France) at 37 °C. Spontaneous ventilation was allowed through an oxygen-delivering mask, with different concentrations of isoflurane depending on the surgical stage (2% during painful stimuli and 1% during latent periods).

At the end of the experiment, left and right gastrocnemius muscles were dissected and immediately immersed in Krebs solution at 4 °C for the extemporaneous analyses of mitochondrial functions. Left and right quadriceps muscles were dissected and immediately immersed in Dulbecco’s phosphate-buffered saline (DPBS) solution, without calcium (Ca^2+^) and magnesium (Mg^2+^) at 4 °C (Sigma-Aldrich, Saint Louis, MI, USA) until muscle dissociation for leucocyte assessment by Fluorescence Activated Cell Sorting (FACS). Left and right gastrocnemius were also dissected and immediately frozen for transcript analysis.

### 4.3. Leucocyte Assessment

The entire quadriceps muscle tissue was cut into small pieces of 2–4 mm, incubated with a commercially available enzyme cocktail (Muscle dissociation Kit, MACS, Miltenyi Biotec, Bergisch Gladbach, Germany) and dissociated with a gentle MACS Dissociator (MACS, Miltenyi Biotec, Bergisch Gladbach, Germany) following the manufacturer’s recommendations. The obtained cell suspension was filtrated through a strainer (70 µm) (VWR), resuspended in an ice-cold DPBS solution without Ca^2+^ and Mg^2+^ (Sigma-Aldrich, Saint-Quentin-Fallavier, France) with ethylenediaminetetraacetic acid (EDTA) 3 mM (Sigma-Aldrich, Saint-Quentin-Fallavier, France) and incubated for 20 min with a mouse anti-CD45 fluorochrome-conjugated antibody (PE-anti-CD45, eBioscience, Villebon-Sur-Yvette, France) at 4 °C in the dark with occasional gentle agitation. Leucocytes, identified as CD45-positive cells, were enumerated among the cell suspension by a compact, easy-to-use fluorescence cell analyzer (Muse^TM^ Cell Analyser; Merck Millipore, Molsheim, France).

### 4.4. Mitochondrial Respiration

Muscle oxygen consumption was determined using a high-resolution oxygraph (Oxygraph 2K, Oroboros Instruments, Innsbruck, Austria), in 2 mL of Miro5 + Cr maintained at 37 °C, containing EGTA (0.5 mM), MgCl2 (3 mM), K lactobionate (60 mM), taurine (20 mM), KH2PO4 (10 mM), HEPES (20 mM), sucrose (110 mM), creatine (20 mM), and BSA (1 g/L), as previously reported. Permeabilized fibers were placed in chambers to record the CI leak (non-coupling respiration after the activation of complex I) with glutamate (10 mM) and malate (2.5 mM). Then, OXPHOS CI (maximal oxidative phosphorylation via the complex I activation) was measured under continuous stirring in the presence of a saturating amount of ADP (2 mM) as a phosphate acceptor. Results were expressed as pmol/s/mg wet weight.

### 4.5. Mitochondrial Calcium Retention Capacity (CRC)

CRC of skeletal muscle mitochondria under energized conditions allowed measurement of the mitochondrial permeability transition pore (mPTP) opening. Briefly, permeabilized fibers (5–6 mg wet weight) were incubated at 4 °C for 30 min under stirring in buffer R+ containing KCl (800 mM) to extract myosin, block the calcium uptake by the sarcoplasmic reticulum, and thus allow calcium uptake only by mitochondria. Then, fibers were washed three times for 10 min in CRC buffer (Tris-Base 20 mM, saccharose 150 mM, KCl 50 mM, KH_2_PO_4_ 2 mM, and succinate 5 mM, pH 7.4 at 23 °C) containing bovine serum albumin (2 mg/mL) and ethylene glycol-bis (β-aminoethyl ether)-N,N,N′,N′-tetraacetic acid (EGTA) (5 μM).

Permeabilized fibers were incubated in quartz tanks with continuous stirring at 24 °C in 1 mL of CRC buffer containing a calcium green-5N fluorescent probe (5 µM; excitation 500 nm; emission 530 nm). The reaction started by adding a calcium pulse (20 mM), followed by calcium pulses every 5 min until mitochondrial permeability transition pore (mPTP) opening. After each pulse, a peak of extramitochondrial calcium was recorded, followed by a rapid uptake by mitochondria. When mitochondria reached its maximal calcium load, mPTP opened and mitochondrial calcium was released, inducing a sharp increase in extramitochondrial calcium concentration.

The amount of calcium necessary to trigger the mPTP opening was calculated from a standard curve relating calcium concentrations to the fluorescence of calcium green-5N. At the end of the experiment, muscle fibers were gathered, dehydrated at 150 °C for 15 min, and weighed. Results were expressed as µmol/mg dry weight.

### 4.6. Reactive Oxygen Species Production

#### 4.6.1. Production of Superoxide Anion

Immediately after harvesting, *gastrocnemius* muscles were placed in Krebs solution containing NaCl 99 mM, KCl 4.69 mM, CaCl_2_ 2.5 mM, MgSO_4_ 1.2 mM, NaHCO_3_ 25 mM, KH_2_PO_4_ 1.03 mM, D (+) Glucose 5.6 mM, Na-Hepes 20 mM, 25 µM of deferoxamine 25 µM and DETC 5 µM. Tissues were cut into 1 to 2 mm^3^ slices.

Because of the short-lived nature of ROS, we used a stable spin label to assess oxidative stress in muscles: 1-hydroxy-3-methoxycarbonyl-2, 2, 5, 5-tetramethylpyrrolidine HCl (CMH). Muscle fragments were incubated for 30 min in Krebs and CMH (200 µM) in a thermo-regulated incubator (37 °C) under controlled pressure (20 mmHg) and gas mix (N_2_: 97.8%, O_2_: 2.8%) to mimic tissue environment (Noxygen^®^, Elzach, Germany). ROS concentrations were then determined with EPR spectroscopy (Bruker Win-EPR^®^, Bruker Analytik GmbH, Ettlingen, Germany) in 40 µL aliquots from muscle fiber supernatants, using the following parameters—microwave power: 60 mW, center field: 2 g, field sweep: 60 G, modulation of amplitude: 2.48 G, conversion time 10.24 ms, time constant 40.96 ms. At the end of the experiment, muscle fragments were gathered, dehydrated and weighted. As a result, ROS production was expressed in µmol/min/mg dry weight.

#### 4.6.2. Production of Hydrogen Peroxide

This production was determined with Amplex Red (Sigma-Aldrich, Saint-Quentin-Fallavier, France, 20 µM) and horseradish peroxidase (HRP; 1 U/mL, Sigma-Aldrich, Saint-Quentin-Fallavier, France) in the Oroboros, simultaneously with the mitochondrial respiration. Briefly, H_2_O_2_ reacted in a 1:1 stoichiometry with amplex red and was catalyzed by HRP. A fluorescent compound is produced, resorufin, in a molar equivalent of H_2_O_2_. The results are expressed in pmol/s/mg wet weight (ww).

### 4.7. Muscle RNA Extraction and RT-qPCR Analysis

Total RNA was isolated from gastrocnemius using TRIzol reagent (Invitrogen, Carlsbad, CA, USA) according to the manufacturer’s instruction. RNA was quantified by spectrophotometry (Nanodrop, Thermo Fisher, Illkirch, France). cDNA was synthesized from 1 µg of RNA by reverse transcription using Maxima H Minus cDNA Synthesis Master Mix (Thermo Fisher Scientific, Illkirch, France) according to the manufacturer’s protocol. cDNA was diluted ten times, and quantitative RT-qPCR analysis was performed with QuantStudio 3 Real-Time PCR System (Applied Biosystem, Waltham, MA, USA) using the PowerTrack SYBR Green Master Mix (Applied Biosystem, Waltham, MA, USA) according to the supplier’s protocol. Primers are described below (Table 1). For each sample, the relative abundance of the transcripts of a given gene was normalized to those of a housekeeping gene (36B4), and data were analyzed using the ∆∆CT methods [52].

### 4.8. Statistical Analysis

All results were expressed as means ± SEM. Data were analyzed using Prism software (GraphPad Prism 8, Graph Pad Software, Inc., San Diego, CA, USA). A Shapiro–Wilk test was applied to verify the normality of the results. If the values followed a normal distribution, the differences between more than two groups were assessed using two-way ANOVA test, followed by Tukey’s post-test. If the values did not follow a normal distribution, a Kruskal–Wallis test was applied. Data were considered to be statistically significant if *p* < 0.05.

## 5. Limitations of the Study and Conclusions

The question of whether reducing the inflammatory response after IR is beneficial is an issue since studies indicate that inflammatory response is important and necessary for proper regeneration. The same interrogation applies when considering oxidative stress. As far as we know, both the degree and the timing of these responses are important. Thus, to be beneficial, both inflammatory and antioxidant responses should be relatively moderate in terms of quantity and duration. In this view, further kinetic studies including several time points with histological analysis will be useful to better characterize the potential beneficial effects of PDE 4 inhibition on IR-related deleterious effects on skeletal muscle.

We can nevertheless conclude that lower-limb ischemia–reperfusion significantly increased muscles leucocytes recruitment and apoptosis, in association with enhanced mitochondrial fission and decreased mitochondrial respiration. PDE 4 inhibition with Rolipram reduced leucocyte infiltration, apoptosis and mitochondrial fission but failed to protect skeletal muscle mitochondrial respiration. Although suggesting partial protection, these data support further studies investigating the usefulness of PDE 4 inhibition during muscle ischemia–reperfusion and whether muscle-specific inhibitors and/or use of compounds resolving an exaggerated inflammation phase [53] might allow enhanced protection.

## Figures and Tables

**Table 1:** Sequence of genes-specific primers used for quantitative polymerase chain reaction.

Gene Name	Primer Forward	Primer Reverse
**36B4**	5′-GAGGAATCAGATGAGGATATGGGA-3′	5′-AAGCAGGCTGACTTGGTTGC-3′
**Bax**	5′-GCCCAGGGACATATCTGACTTC-3′	5′-TCATTGCTGGTGGCTCTCAC-3′
**Bcl2**	5′-CTGAGTACCTGAACCGGCAT-3′	5′-GGTATGCACCCAGAGTGATG-3′
**SOD1**	5′-GGCTTCTCGTCTTGCTCTCTC-3′	5′-AACACAACTGGTTCACCGCT-3′
**SOD2**	5′-CCCAAAGGAGAGTTGCTGGA-3′	5′-TCTGTAAGCGACCTTGCTCC-3′
**Catalase**	5′-CACTGACGAGATGGCACACT-3′	5′-TGTGGAGAATCGAACGGCAA-3′
**Drp1**	5′-AGAAAACTGTCTGCCCGAGA-3′	5′-GCTGCCCTACCAGTTCACTC-3′
**Fis1**	5′-CCGGCTCAAGGAATATGAAA-3′	5′-ACAGCCAGTCCAATGAGTCC-3′
**Mfn1**	5′-CCTCCATGGGCATCATCGTT-3′	5′-TGCAGCTTCTCGGTTGCATA-3′
**Mfn2**	5′-CTCAGGAGCAGCGGGTTTAT-3′	5′-GAGAGGCGCCTGATCTCTTC-3′

## Data Availability

The original contributions presented in this study are included in the article. Further inquiries can be directed to the corresponding author.
